# GRK phosphorylation drives β-arrestin–independent internalization of chemokine receptor CXCR5

**DOI:** 10.1016/j.jbc.2025.111114

**Published:** 2025-12-29

**Authors:** Joseph M. Crecelius, Ya Zhuo, Aaren R. Manz, Julia Drube, Stefan Schulz, Carsten Hoffmann, Adriano Marchese

**Affiliations:** 1 Department of Biochemistry, Medical College of Wisconsin, Milwaukee, Wisconsin, USA; 2 Institut für Molekulare Zellbiologie, CMB – Center for Molecular Biomedicine, Universitätsklinikum Jena, Friedrich-Schiller-Universität Jena, Jena, Germany; 3 Department of Pharmacology and Toxicology, Jena University Hospital, Jena, Germany; 4 7TM Antibodies, Jena, Germany

**Keywords:** BRET, arrestin, GRK, internalization, desensitization, phosphorylation, chemokine receptor

## Abstract

G protein–coupled receptor kinases (GRKs) and β-arrestins act in concert to regulate G protein–coupled receptor signaling and trafficking. Previously, we showed that β-arrestins are essential for desensitization, but not internalization, of the chemokine receptor CXCR5. Here, we investigated the role of GRKs on β-arrestin recruitment, phosphorylation, and internalization of CXCR5 using gene-edited HEK293 cells in which the ubiquitously expressed GRKs have been deleted (GRK2/3/5/6; ΔQ-GRK). Using novel phospho-site–specific antibodies, we demonstrate that CXCL13 stimulation promotes rapid and sustained phosphorylation of the carboxyl terminal region (C-tail) of CXCR5 at paired Ser^367^Thr^368^ and Ser^370^Ser^371^ residues, which we have previously shown are essential for β-arrestin recruitment. Using ΔQ-GRK HEK293 cells coupled with individual GRK2 or GRK5 re-expression, we show that phosphorylation of these residues was rescued by either GRK2 or GRK5, while rescue of agonist-stimulated β-arrestin recruitment showed a preference for GRK2 over GRK5, suggesting that additional phospho-sites are likely involved in β-arrestin recruitment. Extension of these studies revealed that agonist-stimulated internalization of CXCR5 was significantly reduced in ΔQ-GRK HEK293 cells and that GRK2 or GRK5 equally rescued the internalization of WT or phospho-site variants, indicating GRK isoform redundancy in CXCR5 internalization. Further, we show that siRNA-mediated knockdown of clathrin significantly reduced CXCR5 internalization, suggesting that internalization of CXCR5 is *via* clathrin-mediated endocytosis. Taken together, these results reveal that GRKs regulate CXCR5 desensitization and internalization and that internalization occurs through an atypical mode that is β-arrestin–independent and requires GRK phosphorylation of the C-tail.

Classically, G protein–coupled receptor (GPCR) kinases (GRKs) and β-arrestins (β-arrestin-1 and β-arrestin-2, a.k.a. arrestin-2 and arrestin-3, respectively) act in concert to regulate GPCR signaling (1). Agonist activation of GPCRs leads to the recruitment of GRKs that phosphorylate intracellular Ser/Thr residues on the carboxyl terminal region (C-tail) and/or intracellular loops (2). These phosphates trigger the recruitment and binding of β-arrestins, which prevents G protein coupling and facilitates desensitization of GPCR signaling (2, 3). β-arrestins also mediate GPCR internalization *via* clathrin-coated pits by interacting with clathrin and adaptor protein complex-2, which are key components of the endocytic machinery (1). However, in addition to this classical mode of regulation, there are atypical modes of GPCR regulation, suggesting that GPCR regulation is far more complex than can be explained by current concepts (4, 5). Particularly, agonist-stimulated internalization of several GPCRs occurs independently of β-arrestins, although GRKs are involved (6). This diversity in GPCR regulation remains largely understudied.

There is a growing cohort of GPCRs for which β-arrestins are not essential for agonist-stimulated internalization, while being required for other β-arrestin–mediated functions such as desensitization and/or signaling (5, 7, 8, 9). Yet GRK-stimulated phosphorylation of the GPCR is still required for receptor internalization (5, 8). We recently reported that β-arrestins are not essential for agonist-stimulated internalization of the C-X-C chemokine receptor 5 (CXCR5), despite being required for desensitization (4). The precise reason for the divergence in function between β-arrestins and GRKs remains unknown, although for CXCR5, this may be related to the phospho-determinants in the C-tail region of the receptor (4). The C-tail region has three potential phospho-site clusters (proximal, medial, distal), and we determined that the cluster located at the extreme distal region of the C-tail is necessary for agonist-stimulated β-arrestin recruitment, which is likely responsible for desensitization (4). The determinants for internalization have yet to be clearly determined, although mutating two of the three phospho-clusters together reduces internalization, suggesting the phospho-site clusters are redundant for internalization, although to what extent or whether other intracellular regions in addition to the C-tail are required for internalization remains to be determined (4). Whether serine or threonine residues within the phospho-site clusters are indeed phosphorylated also remains unknown.

GRKs belong to a subfamily of AGC kinases comprised of seven (GRKs 1–7) members within the human genome (10, 11, 12). GRK1 and 7 are primarily expressed in the visual system (13). GRK4 is primarily expressed in the gonads, while the remaining GRK2/3/5/6 are ubiquitously expressed (14). GRK isoforms have been shown to phosphorylate multiple phospho-sites upon GPCR activation (15, 16) and this divergence in phospho-site specificity can differ among various GPCRs, which adds to the complexity of GRK regulation of GPCR signaling (8, 17, 18, 19, 20, 21). Adding to this complexity is the fact that GRKs can mediate downstream signaling, independent of their activity of direct phosphorylation of GPCRs (17, 22). Recent studies with genetically modified cells in which each of GRK subfamily (GRK2/3 or GRK5/6) or all (GRK2/3/5/6) have been deleted from HEK293 cells have provided evidence of the roles of these kinases on β-arrestin recruitment and activation and enabled studies for further defining the functional role of GRKs in GPCR signaling and trafficking (8, 19). The role of GRKs in the regulation of CXCR5 signaling and trafficking remains unexplored.

In this present study, we address the role of GRKs on phosphorylation, desensitization, and internalization of the chemokine receptor CXCR5. Using custom-designed phospho-specific antibodies, we show that agonist stimulation promotes rapid and sustained phosphorylation of paired sites Thr367/Ser368 and Thr370/Thr371 located at the extreme distal region of the C-tail. We previously showed that these sites are essential for β-arrestin recruitment and desensitization and here establish that Thr367/Ser368 and Thr370/Thr371 are phosphorylated by GRKs belonging to GRK2 and GRK5 subfamilies using gene-edited HEK293 cells that have been deleted of the four ubiquitously expressed GRKs (GRK2/3/5/6, ΔQ-GRK) (19). Using bystander bioluminescence resonance energy transfer (BRET) and whole-cell ELISA, we provide evidence that GRKs are necessary and sufficient for CXCR5 internalization and the phospho-site determinants for internalization reside within the C-tail of the receptor. We also establish a role for clathrin in CXCR5 internalization.

## Results

**Figure 1:**
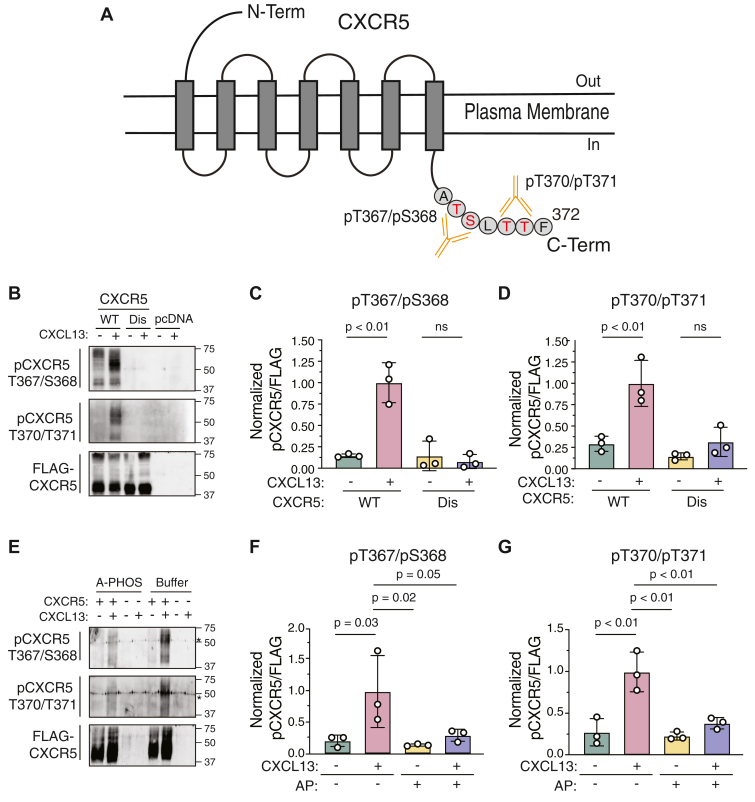
**CXCR5 is phosphorylated at paired Thr^367^Ser^368^ and Thr^370^/Thr^371^ amino acid residues.***A*, diagram showing the location of the anti-pThr^367^/pSer^368^ and anti-pThr^370^/pThr^371^ antibodies at the CXCR5 extreme distal carboxy-terminal tail. The relevant Ser/The residues are in *red* text. *B*, HEK293 cells transiently expressing FLAG-tagged CXCR5 (WT), distal mutant (Dis; Thr^367^Ser^368^, Thr^370^Thr^371^ substituted with alanine), or empty vector (pcDNA) were stimulated without (−) or with 100 nM CXCL13 (+) for 5 min, followed by anti-FLAG immunoprecipitation and immunoblotting with the indicated antibodies. Representative immunoblots from three independent experiments are shown. *C* and *D*, densitometric analysis of (*B*) to quantify receptor phosphorylation. Values for phosphorylation were normalized to receptor levels (FLAG-CXCR5) and then compared to the CXCL13-stimulated WT receptor condition. *E*, alkaline phosphatase (A-PHOS) reduces the detection of CXCR5 phosphorylation. HEK293 cells transiently expressing FLAG-CXCR5 (+) or pcDNA (−) were stimulated without (−) or with (+) 100 nM CXCL13 for 5 min, followed by anti-FLAG immunoprecipitation and treatment without (−) or with (+) alkaline phosphatase (20 units) for 3 h at 37 °C and then immunoblotting with the indicated antibodies. *F* and *G*, densitometric analysis of (*E*) to quantify receptor phosphorylation. Values for phosphorylation were normalized to receptor levels (FLAG-CXCR5) and then compared with CXCL13 stimulated (+/−) no A-Phos condition with CXCR5. Data represents the mean ± S.D. from three independent experiments. Data were analyzed by one-way ANOVA followed by Dunnett’s multiple comparisons test. *p* values lower than 0.05 were considered significant. ∗ = nonspecific band, ns = not significant. CXCR5, C-X-C chemokine receptor 5.

We recently identified a key putative phospho-site cluster on the extreme distal portion of the C-tail of CXCR5 required for β-arrestin–dependent recruitment and desensitization (4). This phospho-site cluster is comprised of two pairs of potential phospho-acceptor residues (T367/S368 and T370/T371) (Fig. 1*A*) and here we used two phospho-antibodies against each pair of phospho-sites to examine their phosphorylation status by CXCL13 stimulation. HEK293 cells transiently expressing FLAG-tagged WT CXCR5 or a distal phospho-site receptor variant (Dis; T367/S368/T370/T371 were substituted for Ala) or empty vector (pcDNA) were stimulated with CXCL13 (100 nM) for 30 min followed by immunoprecipitation and immunoblotting with the phospho-site antibodies. Increased immunoreactivity of both phospho-site antibodies was detected with the WT receptor from cells stimulated with CXCL13 compared to vehicle, while no immunoreactivity was detected with the Dis variant or vector control (Fig. 1*B* and quantified in 1C/D), suggesting the specificity of the phospho-site antibodies. The phosphorylated receptor appeared as a broad band, which is similar to what is observed with the anti-FLAG immunoblot of the immunoprecipitated receptor. The broad band appearance of the receptor is likely due to other posttranslational modifications, such as glycosylation, which has been observed in other GPCRs (23). Importantly, immunoreactivity was reduced from samples treated with alkaline phosphatase to remove phosphates, further indicating the antibodies are detecting phosphorylated receptor (Fig. 1*E* and quantified in 1*F*/*G*). Overall, these results confirm that these novel phospho-site specific antibodies are indeed detecting the phosphorylation of T367/S368 and T370/T371 on CXCR5.

**Figure 2:**
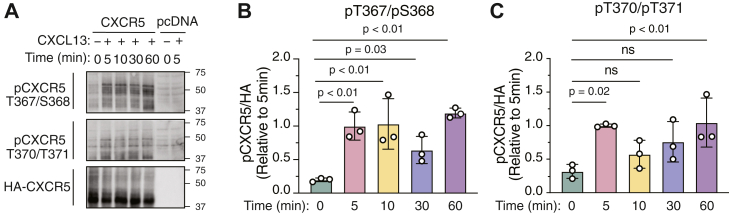
**Time-dependent analysis of agonist-stimulated phosphorylation of CXCR5.***A*, HEK293 cells transiently expressing HA-tagged CXCR5 (WT) or empty vector (pcDNA) were stimulated without (−) or with 100 nM CXCL13 (+) for 5 to 60 min, followed by anti-HA immunoprecipitation and immunoblotting with the indicated antibodies. Representative immunoblots are shown. *B* and *C*, densitometric analysis of (*A*) to quantify receptor phosphorylation. Values for phosphorylation were normalized to receptor levels (HA-CXCR5) and then compared with vehicle (−) condition. Data represent the mean ± S.D. from three independent experiments. Data were analyzed by two-way ANOVA followed by Dunnett’s multiple comparisons test. *p* values are indicated and values < 0.05 were considered significant. ns = not significant. CXCR5, C-X-C chemokine receptor 5.

We evaluated the kinetics of phosphorylation by stimulating cells transiently expressing HA-CXCR5 for various times with 100 nM CXCL13. Phosphorylation of both phospho-pairs occurred rapidly, within 5 min of CXCL13 stimulation, and remained sustained for the duration of the 60 min stimulation (Fig. 2*A* and quantified in 2, *B* and *C*). Taken together, we have defined two site-specific phospho-antibodies and established for the first time that T367/S368 and T370/T371 are rapidly phosphorylated following agonist stimulation.

**Figure 3:**
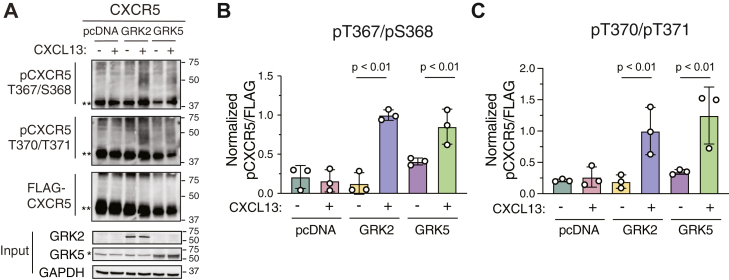
**Phosphorylation of CXCR5 is restored by the re-expression of GRK2 and GRK5 in ΔQ-GRK HEK293 cells.***A*, ΔQ-GRK HEK293 cells transiently expressing either FLAG-CXCR5 (n = 1, 2), HA-CXCR5 (n = 3), and pcDNA, GRK2, or GRK5 were stimulated for 10 min without (−) or with (+) 100 nM CXCL13, followed by anti-HA or anti-FLAG immunoprecipitation and immunoblotting with the indicated antibodies. Representative immunoblots are shown. Two asterisks (∗∗) represents unmodified receptor, while one asterisk (∗) represents a nonspecific band. *B* and *C*, densitometric analysis of (A) to quantify receptor phosphorylation. Values for phosphorylation were normalized to receptor levels immunoprecipitated (FLAG-CXCR5 or HA-CXCR5) and then compared with the GRK2 stimulated (+) condition. Data represent the mean ± S.D. from three independent experiments. Data were analyzed by one-way ANOVA followed by Šídák's multiple comparisons test. *p* values are indicated. GRK, G protein–coupled receptor kinase; CXCR5, C-X-C chemokine receptor 5.

GPCRs are typically phosphorylated by GRKs, a family of kinases that selectively recognize agonist-stimulated and agonist-occupied GPCRs (10). To address the potential role of GRKs, we examined CXCR5 phosphorylation using a HEK293 cell line (ΔQ-GRK) genetically deleted the four widely expressed GRK isoforms (GRK2/3/5/6) (19). CXCL13-stimulated phosphorylation of both phospho-pairs was absent in empty vector (pcDNA) expressing cells, consistent with these residues being phosphorylated by GRKs (Fig. 3*A*). When GRK2 or GRK5 were re-expressed, phosphorylation of both phospho-pairs was restored (Fig. 3*A* and quantified in 3, *B* and *C*), indicating the importance of these GRKs in the phosphorylation of T367/S368 and T370/T371, suggesting that there is no GRK isoform specificity at these sites. However, other quantitative approaches would be necessary to precisely define the relative contributions of GRK2 and GRK5 to the phosphorylation of these sites.

**Figure 4:**
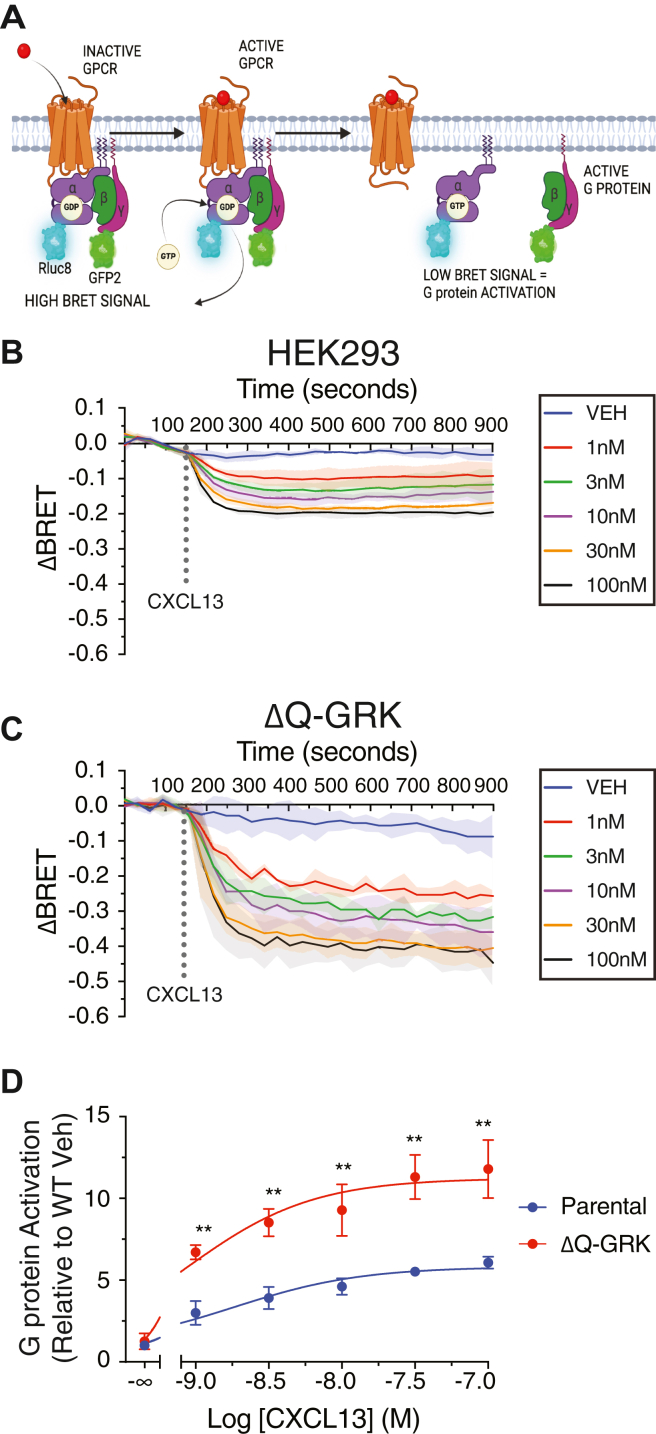
**CXCR5-stimulated G protein activation in WT or ΔQ-GRK HEK293 cells.***A*, TRUEPATH model figure; donor tagged (Rluc8) G_α_, Gβ, Gγ-tagged acceptor tagged (GFP2) subunits are transfected at equal DNA ratios. BRET signal starts high in the inactivated (GDP) bound state; GPCR activation then promotes a decrease in BRET signal as the G_α_ and Gβγ subunits separate when GDP is exchanged for GTP. *B* and *C*, WT (*B*) and ΔQ-GRK HEK293 cell lines (*C*) were transiently transfected with FLAG-tagged CXCR5, G_i1_-Rluc8, β_3_, and γ_9_-GFP2. Baseline BRET signals were measured for 150 s, followed by the manual addition of varying concentrations of CXCL13 (1–100 nM), and the BRET signal was measured every 30 s for 15 min. The average BRET signal of the baseline was subtracted from the BRET of the CXCL13 response and normalized to receptor expression (ΔBRET). Data are the mean ± S.D. of three independent experiments. *D*, dose response relationships calculated from the area under the curve (AUC) analysis of data shown in (*B* and *C*). AUC values were normalized to the HEK293 vehicle condition. Data are the mean ± S.D. of three independent experiments. Where not visible, the error bars fall within the symbol. Data were analyzed by two-way ANOVA followed by Šídák's multiple comparisons test. Asterisks (∗∗) represent *p* < 0.01. GPCR, G protein–coupled receptor; GRK, G protein–coupled receptor kinase; CXCR5, C-X-C chemokine receptor 5; BRET, bioluminescence resonance energy transfer.

Previously, we showed that β-arrestins are required for CXCR5 desensitization (4), but the role of GRKs remains unknown. To address this, we measured heterotrimeric G protein (Gαβγ) activation of agonist-stimulated CXCR5 using TRUEPATH in WT and ΔQ-GRK HEK293 cells (Fig. 4*A*). In this BRET-based approach, heterotrimer Gαβγ dissociation is measured as a loss in the BRET signal between donor G⍺-RLuc8 and acceptor Gβ*γ*-GFP^2^ (24). Each G protein subunit (Gαβγ) is transiently cotransfected into cells in equal amounts along with FLAG-CXCR5, and CXCL13-stimulated BRET responses are measured over time and normalized to receptor levels as assessed in parallel by ELISA. In WT HEK293 cells, CXCL13 promoted a rapid concentration-dependent reduction in the BRET response, consistent with receptor-dependent heterotrimer dissociation and activation (Fig. 4*B*). Similarly, CXCL13 promoted a rapid reduction in the BRET response in ΔQ-GRK HEK293 cells; however, the magnitude was much larger than that observed in WT HEK293 cells (Fig. 4*C*). We quantified this effect by calculating the area under the curve (AUC) at each concentration of CXCL13 and normalized these values to the vehicle condition from the WT HEK293 cells (Fig. 4*D*). The potency (EC50) is somewhat higher (∼1.2 fold) in ΔQ-GRK cells (8.5 × 10^−10^ M) than WT cells (1 × 10^−9^ M), and the efficacy is 2-fold larger in the ΔQ-GRK cells than in WT cells (Fig. 4*D*). Overall, these data are consistent with enhanced CXCR5 coupling to heterotrimeric G proteins in the ΔQ-GRK HEK293 cell line, consistent with GRKs being required for CXCR5 desensitization.

**Figure 5:**
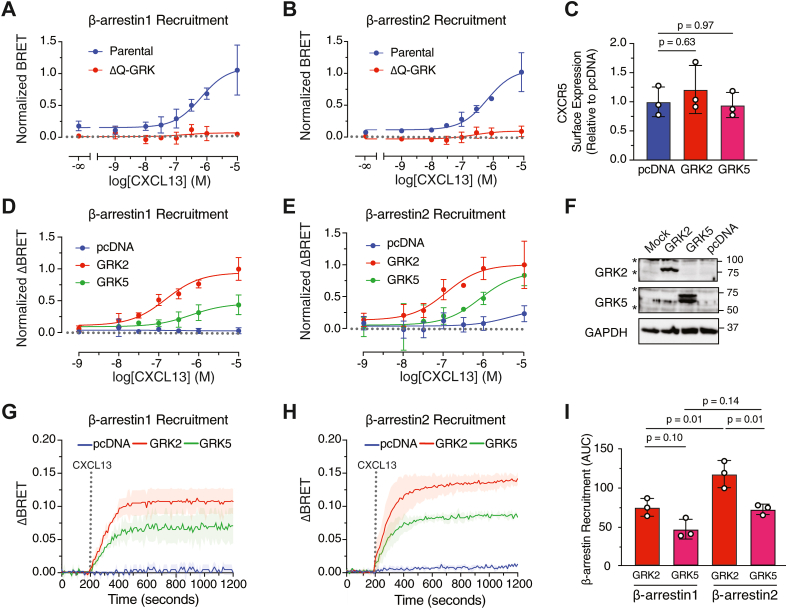
**GRKs are essential for β-arrestin recruitment to CXCR5.***A* and *B*, WT and ΔQ-GRK HEK293 cell lines transiently expressing HA- and RLuc8-tagged CXCR5 (HA-CXCR5-Rluc8) and pcDNA (background), β-arrestin1-GFP^10^ (*A*) or β-arrestin2-GFP^10^ (*B*) were stimulated with varying concentrations of CXCL13. BRET signal is representative of three consecutive reads every 30 s averaged together with background (pcDNA) condition subtracted. AUC values were normalized to the highest concentration of CXCL13 (10 μM) from the parental HEK293 cells. Data represent the mean ± S.D. of three independent experiments. Where not visible, the error bars fall within the symbol. Data were fit using GraphPad prism nonlinear regression model [log[agonist]vs. response (three parameters)]. *C*, surface expression of FLAG-tagged CXCR5 in ΔQ-GRK HEK293 cells transfected with empty vector (pcDNA), GRK2, or GRK5 normalized to pcDNA condition. *D* and *E*, cells were transfected with β-arrestin1-GFP^10^ (*D*) or β-arrestin2-GFP^10^ (*E*) and BRET signals were measured as in (*A*) and (*B*) and the BRET value for the vehicle treatment of each transfection condition was subtracted from each CXCL13 dose. Data represent the mean ± S.D. of three independent experiments. Where not visible, the error bars fall within the symbol. Data were fit using GraphPad prism nonlinear regression model [log[agonist]vs. response (three parameters)]. *F*, representative immunoblots with indicated antibodies. *G*–*I*, kinetic profile of β-arrestin recruitment to CXCR5 by bystander recruitment of NLuc-tagged β-arrestin1 (*G*) or β-arrestin2 (*H*) to plasma membrane–targeted Venus (Venus-CAAX). Baseline BRET signals were measured for 200 s, followed by the manual addition of vehicle or CXCL13 (100 nM) and the BRET signal was measured every 10 s for 20 min. The average BRET signal of the baseline was subtracted from the BRET response of each condition. Data were further normalized by subtracting the vehicle values at each time point from the CXCL13-stimulated values in each transfection condition (ΔBRET). *I*, area under the curve (AUC) analysis of data shown in (*G* and *H*). AUC values were normalized to the FLAG-CXCR5 surface expression in each condition, measured by ELISA in parallel with BRET experiments. Data are the mean ± S.D. of three independent experiments. Data were analyzed by one-way ANOVA followed by Tukey’s multiple comparisons test. *p* values are shown; values < 0.05 were considered significant. Asterisks (∗) indicates nonspecific band. GRK, G protein–coupled receptor kinase; CXCR5, C-X-C chemokine receptor 5; BRET, bioluminescence resonance energy transfer.

Previously, we showed that the extreme distal phospho-site cluster is required for β-arrestin recruitment to CXCR5 (4). To address the role of GRKs, we examined β-arrestin-1 (β-arr1) and β-arrestin-2 (β-arr2) recruitment to CXCR5 by BRET following agonist stimulation in parental and ΔQ-GRK HEK293 cells. CXCL13-stimulated β-arr1-GFP^10^ and β-arr2-GFP^10^ recruitment to CXCR5-Rluc8 was completely absent in the ΔQ-GRK HEK293 cells when compared with parental cells (Fig. 5, *A* and *B*). Re-expression of either GRK2 or GRK5 rescued CXCL13-stimulated β-arr1 and β-arr2 recruitment to CXCR5 in the ΔQ-GRK cells (Fig. 5, *C*–*E*). Notably, GRK2 showed greater efficacy at β-arr1 and β-arr2 recruitment over GRK5, suggesting that other receptor determinants beyond the extreme distal region may also play a role in β-arrestin recruitment to CXCR5. Cell surface levels of FLAG-tagged CXCR5 were similar in cells transfected with GRK2 and GRK5 or without pcDNA (Fig. 5*C*). We also verified the expression of GRKs in the ΔQ-GRKs by immunoblotting of samples collected in parallel to the BRET assays (Fig. 5*F*)

We next examined real-time kinetics of agonist-stimulated β-arr1 and β-arr2 recruitment to CXCR5 using a bystander BRET assay. For this, we assessed the BRET signal by NanoLuc luciferase (NLuc)-β-arr1 and NLuc-β-arr2 with plasma membrane–targeted fluorescent protein Venus (PM-Venus) in the ΔQ-GRK HEK293 cells in response to CXCL13 stimulation (Fig. 5, *G* and *H*). The BRET signal was completely absent in the ΔQ-GRK cells consistent with a lack of β-arr1 and β-arr2 recruitment to CXCR5 (Fig. 5, *G* and *H*). When either GRK2 or GRK5 was re-expressed, the BRET signal rapidly increased and peaked within 3 to 4 min of CXCL13 stimulation and was sustained for the 20 min duration of the measurements (Fig. 5, *G* and *H*), consistent with rescue of β-arr1 and β-arr2 recruitment to CXCR5. Interestingly, the peak response of either β-arr1 or β-arr2 with GRK2 re-expression was approximately twice than that observed with GRK5 (Fig. 5*I*), suggesting that divergent receptor determinants are responsible for GRK2- and GRK5-mediated recruitment of β-arrestins to CXCR5.

**Figure 6:**
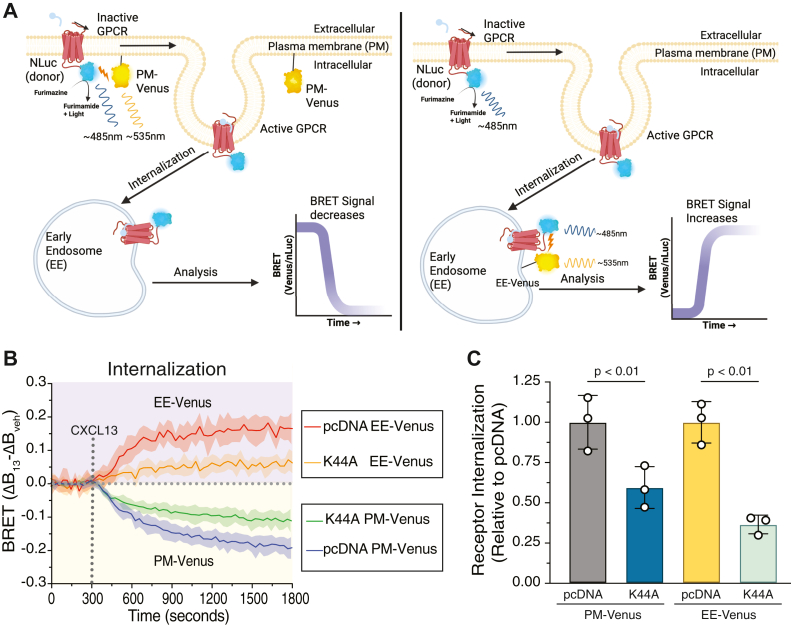
**Real-time measurement of GPCR internalization by bystander BRET.***A*, schematic illustrating bystander BRET to monitor GPCR internalization. *Left Panel:* Upon ligand activation, NLuc (donor)-tagged GPCR (GPCR-NLuc) moves away from the plasma membrane upon internalization and loses proximity to plasma membrane–targeted acceptor protein Venus (PM-Venus) resulting in a decreased BRET signal. *Right Panel:* Upon ligand activation, GPCR-NLuc internalizes onto early endosomes where it comes into proximity of early endosomal-localized acceptor Venus (EE-Venus) resulting in an increased BRET signal. *B*, HEK293 cells were transiently transfected with CXCR5-NLuc and either PM-Venus or EE-Venus plus dominant negative dynamin-K44A or empty vector (pcDNA). Baseline BRET signals were measured for ∼5 min, followed by the manual addition of CXCL13 (100 nM) or vehicle and the BRET signal was measured every 30 s for ∼25 min at 37 °C. The average BRET signal of the baseline was subtracted from the BRET response of CXCL13 or vehicle, followed by subtracting the vehicle BRET from the CXCL13 BRET (ΔBRET). *C*, area under the curve calculations from data in (*B*). PM-Venus and EE-Venus were normalized to their respective pcDNA transfection conditions. Data represent the mean ± S.D. from three independent experiments. Data were analyzed by one-way ANOVA followed by Šídák's multiple comparisons test. *p* values are indicated. GPCR, G protein–coupled receptor; CXCR5, C-X-C chemokine receptor 5; BRET, bioluminescence resonance energy transfer.

**Figure 7:**
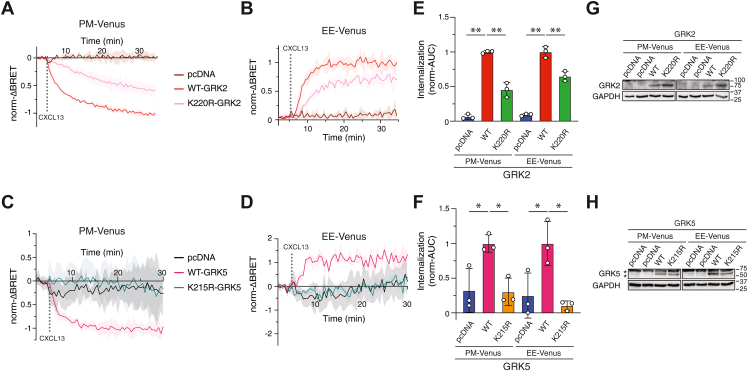
**GRKs are essential for agonist-stimulated internalization of CXCR5.***A*–*E*, ΔQ-GRK HEK293 cells were transiently transfected with FLAG-CXCR5-NLuc and either Venus targeted to the plasma membrane (PM-Venus) or early endosomes (EE-Venus) plus either empty vector (pcDNA), WT GRK2 and GRK5, or kinase-dead mutants of GRK2 (K220R) and GRK5 (K215R). Baseline BRET signals were acquired for 5 min, followed by the manual addition of vehicle or CXCL13 (100 nM) and BRET measurements were recorded every 30 s for 25 to 30 min at 37 °C. The average BRET signal of the baseline was subtracted from the BRET response of CXCL13 or vehicle, followed by subtracting the vehicle BRET from the CXCL13 BRET (ΔBRET). ΔBRET was normalized to the maximum response of WT GRK2 or GRK5 (norm-ΔBRET). Data represent the mean ± S.D. of three independent experiments. *E* and *F*, area under the curve (AUC) analysis. AUC values were normalized to their respective WT condition. Data represent the mean ± S.D. of three independent experiments. Data were analyzed by one-way ANOVA followed by Dunnett’s multiple comparison test. Asterisks represent adjusted *p* values: ∗, *p* < 0.05; ∗∗, *p* < 0.001. *G* and *H*, representative immunoblots of GRK2 and GRK5 expression. Asterisk (∗) represents, nonspecific band (*H*). GRK, G protein–coupled receptor kinase; CXCR5, C-X-C chemokine receptor 5; BRET, bioluminescence resonance energy transfer.

We previously reported that β-arrestins are not essential for agonist-stimulated internalization of CXCR5; however, the role of GRKs remains to be determined (4). To examine the role of GRKs on agonist-stimulated CXCR5 internalization, we employed a BRET-based “bystander” or proximity assay to measure CXCR5 internalization in real-time under acute and selective pharmacological inhibition of GRK2 or GRK5. In this assay, a decrease in the BRET signal from baseline between CXCR5-NLuc and plasma membrane–targeted Venus (PM-Venus) indicates receptor internalization from the plasma membrane and an increase in the BRET signal from baseline between CXCR5-Nluc and early endosome-targeted Venus (EE-Venus) indicates internalization onto early endosomes (25) (Fig. 6*A*). We first confirmed that this assay accurately measures CXCR5 internalization. After CXCL13 activation, the BRET signal between CXCR5-NLuc and PM-Venus rapidly decreased from baseline and continued to decrease slowly for the duration of the stimulation, consistent with removal of the receptor from the plasma membrane or internalization (Fig. 6*B*, bottom panel). This decrease correlated with a rapid and sustained increase in the BRET signal between CXCR5-NLuc and EE-Venus, which is consistent with the appearance of CXCR5 on early endosomes after internalization (Fig. 6*B*, top panel). In cells expressing a dominant negative variant (K44A) of dynamin, which we have previously shown that agonist-stimulated internalization of CXCR5 is *via* a dynamin-dependent pathway (26), the BRET signal was significantly reduced between CXCR5-NLuc and PM-Venus or EE-Venus (Fig. 7*B* and quantified in 7*C*), consistent with inhibition of CXCR5 internalization. Therefore, we conclude that this assay provides a reliable and accurate measure of CXCR5 internalization.

We next used this assay to examine CXCL13-stimulated internalization of CXCR5 in ΔQ-GRK cells. In empty vector (pcDNA) transfected cells, the BRET signal remained unchanged from baseline after the addition of CXCL13 between CXCR5-NLuc and either PM-Venus (Fig. 7, *A*, *C*, *E*, *F*) or EE-Venus (Fig. 7, *B*, *D*, *E*, *F*), consistent with CXCR5 internalization being essentially abolished in the ΔQ-GRK cells. However, when GRK2 and GRK5 were re-expressed, the BRET signal robustly changed from baseline after the addition of CXCL13, indicating effective restoration of CXCR5 internalization (Fig. 7, *A*–*F*), likely *via* dynamin-dependent pathway, although this remains to be examined in the rescue experiments. These results also indicate that the loss of CXCR5 internalization in the ΔQ-GRK cells is not due to a clonal cell line defect and is instead due to a loss of GRKs. Because GRKs may scaffold other proteins independent of their kinase activity to mediate GPCR internalization (27), such as binding to clathrin-heavy chain (28), we examined the expression of “kinase-dead” variants of GRK2 and GRK5 (GRK2-K220R; GRK5-K215R) (29) on CXCR5 internalization. The “kinase-dead” variant of GRK2 only partially rescued CXCR5 internalization (Fig. 7, *A* and *B*, *E*), while the GRK5 variant failed to rescue CXCR5 internalization (Fig. 7, *C* and *D*, *F*). Samples were collected in parallel to confirm GRK expression by immunoblotting (Fig. 7, *G* and *H*). These results suggest that receptor phosphorylation and not a scaffolding role of GRKs is essential for CXCR5 internalization.

**Figure 8:**
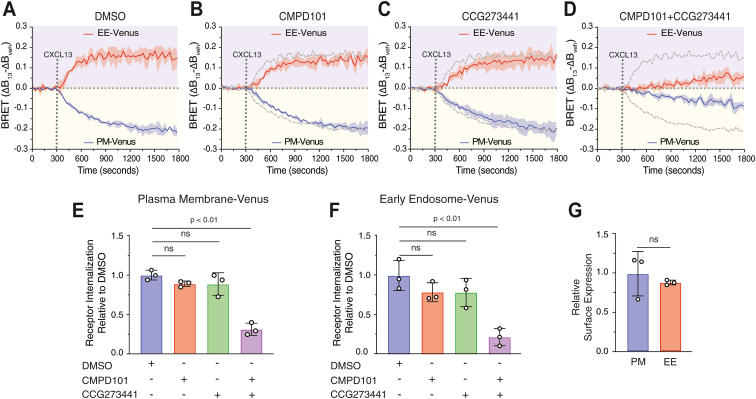
**Analysis of CXCR5 internalization under acute pharmacological inhibition of GRK2 and GRK5.***A*–*D*, HEK293 cells were transiently transfected with FLAG-tagged CXCR5-NLuc and either Venus targeted to the plasma membrane (PM-Venus) or early endosomes (EE-Venus). Cells were pretreated with vehicle (DMSO), 10 μM CMPD101 (GRK2/3 inhibitor), or 10 μM CCG273441 (GRK5/6 inhibitor) kinase inhibitors for 15 min at 37 °C before acquiring baseline BRET signals for 5 min, followed by the manual addition of vehicle or CXCL13 (100 nM) and BRET measurements every 30 s for 25 min at 37 °C. The average BRET signal of the baseline was subtracted from the BRET response of CXCL13 or vehicle, followed by subtracting the vehicle BRET from the CXCL13 BRET (ΔBRET). The faint *gray dashed line* represents the DMSO control (*A*). Data represent the mean ± S.D. of three independent experiments. *E* and *F*, area under the curve (AUC) analysis. AUC values were normalized to their respective DMSO conditions. Data were analyzed by one-way ANOVA followed by Dunnett’s multiple comparison test. *G*, surface expression of FLAG-tagged CXCR5-NLuc in each transfection condition was measured by parallel ELISA and compared using an unpaired Student’s *t* test. *p* values are indicated and ns represents not significant. GRK, G protein–coupled receptor kinase; CXCR5, C-X-C chemokine receptor 5; BRET, bioluminescence resonance energy transfer.

We next examined the effect of acute pharmacological inhibition of GRK2 and GRK5 on CXCL13-stimulated CXCR5 internalization. For pharmacological inhibition, we used compound 101 (CMPD101), which selectively inhibits the GRK2/3 subfamily, and compound CCG273441, which selectively inhibits the GRK5/6 subfamily (30, 31). When given alone, each inhibitor only mildly reduced the BRET signal between CXCR5-NLuc and either PM-Venus or EE-Venus when compared with the DMSO control (Fig. 8, *A*–*C* and quantified in 8E/F). However, when given together, the BRET signal was significantly reduced, consistent with the inhibition of CXCR5 internalization (Fig. 8*D* and quantified in Fig. 8, *E* and *F*). The cell surface expression of FLAG-tagged CXCR5-NLuc from parallel samples from three independent biological replicates was similar (Fig. 8*G*). Overall, these data are consistent with data from Figure 7, in which we show that either GRK2 or GRK5 re-expression rescues CXCR5 internalization in the ΔQ-GRK HEK293 cell line. These results indicate functional redundancy among GRKs in mediating CXCR5 internalization. This redundancy may involve the phosphorylation of distinct, nonoverlapping sites, consistent with the view that GRKs target discrete phosphorylation sites on GPCRs (14).

**Figure 9:**
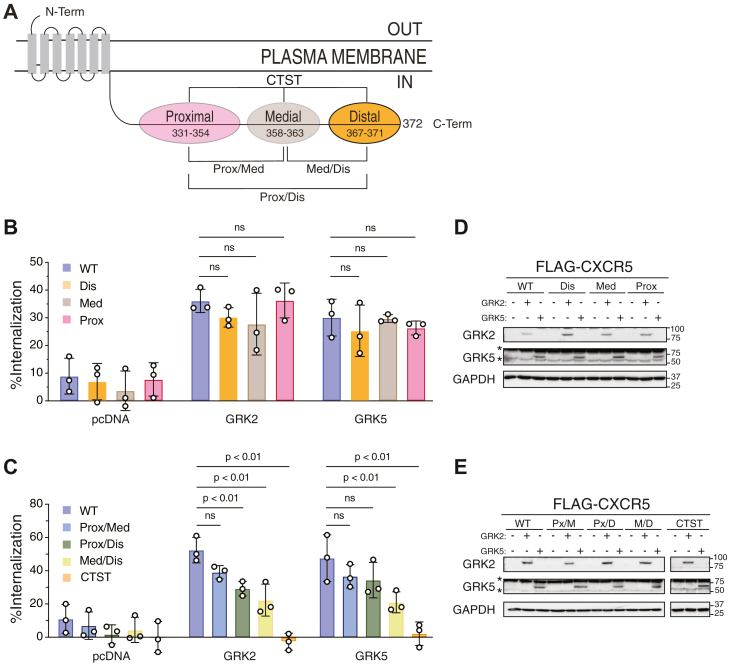
**Analysis of GRK2 and GRK5 re-expression on the internalization of CXCR5 phospho-site cluster variants in ΔQ-GRK HEK293 cells.***A*, schematic representation of the carboxyl-terminal tail of CXCR5 with the designated phospho-site clusters. Double cluster variants and the triple cluster variant (CTST) are indicated. The amino acid range of each cluster is indicated. Ser/Thr residues are mutated to Ala within each cluster. *B* and *C*, ΔQ-GRK cells transiently expressing FLAG-tagged WT CXCR5 and single cluster variants (Dis; Med, Prox) (*B*) or double cluster (Prox/Med; Prox/Dis; Med/Dis) and triple cluster (CTST) variants (*C*), plus empty vector (pcDNA), GRK2, or GRK5. Cells were stimulated with vehicle or 100 nM CXCL13 for 30 min at 37 °C and cell surface receptor was measured by ELISA, as described in Experimental procedures. Internalization was calculated as a percent decrease in the background-adjusted absorbance values from CXCL13-stimulated cells relative to vehicle-treated cells. Data represent the mean ± S.D. from three independent experiments. Data were analyzed with one-way ANOVA with Dunnett’s multiple comparisons comparing the internalization of GRK2 or GRK5 add-back between the pcDNA of its respective CXCR5 variant. *p* values are indicated, and ns denotes not significant. *D* and *E*, representative immunoblots with the indicated antibodies are shown. Asterisk (∗) represents, nonspecific band. GRK, G protein–coupled receptor kinase; CXCR5, C-X-C chemokine receptor 5.

GRK isoforms have been shown to phosphorylate discrete Ser/Thr residues on GPCRs (17, 22, 32, 33). In addition to the extreme distal phospho-site cluster (Fig. 1), there are medial and proximal phospho-site clusters along the C-tail of CXCR5 (Fig. 9*A*). We have previously shown that single or double phospho-site cluster variants were minimally impacted when assessed for agonist-stimulated internalization, suggesting that these phospho-clusters act in a redundant fashion (4). To explore this further, we examined internalization by ELISA in the ΔQ-GRK cells expressing each of these receptor variants, including a new variant in which all three phospho-clusters have been substituted for alanine residues (CTST). We first examined the single-cluster variants (Prox, Med, Dis) in which two of the phospho-site clusters remains intact. Re-expression of GRK2 or GRK5 rescued receptor internalization to the same level for each variant as WT receptor, consistent with redundancy of each phospho-site cluster and GRK isoform (Fig. 9*B*). We next examined internalization of the double-phospho-site cluster variants (Prox/Med, Prox/Dis, Med/Dis) in which only one of the phospho-site clusters remains intact. Strikingly, re-expression of either GRK2 or GRK5 rescued internalization of each respective variant to the same level, but each variant showed different levels of rescue (Fig. 9*C*). Specifically, the Med/Dis variant in which the proximal region remains intact showed the least rescue, followed by the Prox/Dis and Prox/Med variants (Fig. 9*C*). These data suggest that the Dis and Med phospho-site clusters play a predominant role in CXCR5 internalization, while the Prox phospho-site cluster plays a less prominent role, although clearly each cluster contributes to receptor internalization. Importantly, substitution of all potential phospho-sites to alanine residues in the C-tail (CTST) completely abolishes receptor internalization, which cannot be rescued by the re-expression of GRK2 and GRK5 (Fig. 9*C*), indicating that C-tail Ser/Thr residues are absolutely necessary for internalization. Whole-cell lysates were collected in parallel for each replicate to visualize GRK re-expression *via* immunoblotting for the single cluster (Fig. 9*D*) and double cluster (Fig. 9*E*) variants.

**Figure 10:**
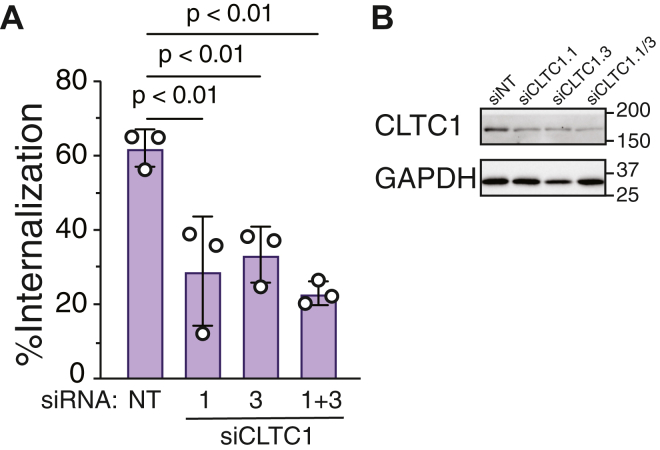
**Role of clathrin on agonist-stimulated internalization of CXCR5.***A*, HEK293 cells in which β-arrestin1 and β-arrestin2 are deleted (DKO) were transiently transfected with FLAG-tagged CXCR5 and siRNA targeting clathrin heavy chain 1 (CLTC1) or a nontargeting siRNA (NT). Cells were stimulated with vehicle or 100 nM CXCL13 for 30 min at 37 °C and cell surface receptor was measured by ELISA, as described in Experimental procedures. Internalization was calculated as a percent decrease in the background-adjusted absorbance values from CXCL13-stimulated cells relative to vehicle-treated cells. Data represent the mean ± S.D. from three independent experiments. Data were analyzed by one-way ANOVA followed by Dunnett’s multiple comparison test. *p* values are indicated. *B*, representative immunoblots for clathrin (CLTC1) and GAPDH control are shown. CXCR5, C-X-C chemokine receptor 5.

GPCRs can internalize *via* clathrin-dependent pathways (34). To determine the primary pathway of CXCR5 internalization, we performed siRNA-mediated knockdown of the clathrin heavy chain 1 (CLTC), which is a major component of clathrin-mediated endocytosis (35, 36). We used HEK293 cells in which β-arrestin-1 and β-arrestin-2 are deleted (double knockout, DKO) for these experiments because they provide a clean background to assess the internalization of CXCR5 independent of β-arrestins. Cells transfected with two discrete siRNA targeting CLTC and CXCL13-stimulated CXCR5 internalization was examined by whole-cell ELISA. We observed that siRNA against CLTC significantly reduced the internalization of CXCR5 when compared with control siRNA (Fig. 10*A*). We confirmed robust knockdown of CLTC by immunoblotting (Fig. 10*B*). These data suggest that CXCR5 mainly follows a clathrin-dependent pathway for internalization.

## Discussion

GRKs and β-arrestins typically act in concert to mediate GPCR desensitization, internalization, and signaling (37, 38). However, it is emerging that β-arrestins are dispensable for the internalization for some GPCRs while being required for desensitization and/or signaling (5, 6, 39). Although β-arrestins are dispensable, GRK-mediated receptor phosphorylation appears to be a prerequisite for internalization, suggesting a distinct mode of internalization for these receptors. We previously reported that β-arrestins were recruited to the chemokine receptor CXCR5 and are only required for receptor desensitization and not internalization (4). Here we provide evidence that CXCR5 phosphorylation by GRKs is required for both desensitization and internalization of CXCR5. Additionally, we provide evidence that CXCR5 internalizes *via* a dynamic-dependent and clathrin-dependent pathway, but the exact mode of endocytosis remains to be determined. This study demonstrates that while β-arrestins are not essential for CXCR5 internalization, GRK-mediated phosphorylation of the C-terminal regions is absolutely required. More broadly, our study contributes to further understanding of the determinants mediating GPCR internalization.

Here, we characterize two phospho-specific antibodies that recognize phosphorylation of two paired residues (pT367/pS368 and pT370/pT371) at the extreme distal C-tail of CXCR5 (Fig. 1). We previously showed these residues are required for β-arrestin recruitment and desensitization of CXCR5 signaling (4). Here, we establish that CXCL13-stimulated CXCR5 activation promotes rapid phosphorylation of each pair of residues (Fig. 2), which matches the kinetics of β-arrestin recruitment (Fig. 5, *G* and *H*). A potential limitation is that given the close proximity of the phospho-pairs within the protein sequence, we cannot rule out the possibility that the two antibodies recognize overlapping epitopes. Further, we cannot conclude that GRK2 and GRK5 equally phosphorylate these phospho-pairs as suggested by the rescue of receptor phosphorylation (Fig. 3). In addition, we cannot rule out the possibility that only one of the residues within the phospho-pair is indeed phosphorylated; other approaches, such as mass spectrometry would be required to determine this. Nevertheless, these phospho-antibodies recognize phosphorylated CXCR5 and may be useful for determining the phosphorylation status of CXCR5 in physiologically relevant cell types and tissues.

We provide evidence that GRK2 and GRK5 have redundant functions at CXCR5. Both GRK2 and GRK5 expression in the ΔQ-GRK KO cells resulted in the rescue of β-arrestin recruitment to CXCR5, although GRK2 resulted in greater rescue than GRK5 (Fig. 5). Because the distal phospho-site cluster, rather than the medial or proximal clusters, preferentially mediates β-arrestin recruitment to CXCR5 (4), this difference in rescue may reflect distinct phosphorylation patterns by GRK2 and GRK5. Although β-arrestins are not essential for CXCR5 internalization (4), we show here that GRKs are essential. Expression of both GRK2 and GRK5 resulted in the rescue of CXCR5 internalization in the ΔQ-GRK KO cells (Fig. 6) and that dual pharmacological inhibition was required to reduce internalization in parental HEK293 cells (Fig. 8), overall supporting redundant functions of GRK2 and GRK5 on internalization. Further, internalization of each phospho-cluster was equally rescued in the ΔQ-GRK KO cells by GRK2 or GRK5 expression, suggesting that each phospho-cluster has a redundant role in internalization (Fig. 9). Notably, while expression of either GRK2 or GRK5 alone or together was sufficient to rescue CXCR5 internalization (Fig. 7 and Supporting Information Fig. S1)), the degree of rescue never achieved the level observed in parental HEK293 cells (∼40 vs 60%, Supporting Information Fig. S1). It is possible that other GRKs may be required for full rescue of internalization and/or show a preference for one of the three phosphor-clusters, which are essential for internalization (Figs. 7 and 9 and Supporting Information Fig. S1 and S2). Alternatively, other non-GRK kinases may also be required for CXCR5 internalization (40). In line with this idea, while internalization was almost completely blocked in the ΔQ-GRK cell line (Figs. 7 and 9), dual inhibition of GRK2/3 and GRK5/6 only reduced CXCR5 internalization by ∼75% (Fig. 8), suggesting that other non-GRK kinases may play a role in CXCR5 internalization. It is likely that multiple kinases act redundantly across several phosphorylation sites to ensure an optimal level of regulation of CXCR5 signaling.

Our study contributes to the growing evidence that GPCR endocytosis can occur through a β-arrestin–independent, GRK-dependent mechanism. Several GPCRs have been recently identified to follow this mode of endocytosis using HEK293 cells deleted of GRKs. For example, β-arrestins are dispensable for internalization of the glucagon-like peptide-1 receptor, while GRKs are essential (8). Similarly, GRKs are essential for FPR2 receptor internalization, while β-arrestins are dispensable (9). Interestingly, agonist-stimulated internalization of the D2 dopamine receptor was only partially reduced in ΔQ-GRK2 cells, suggesting a GRK-independent mode of internalization (41). It is noteworthy that a common feature among these GPCRs and CXCR5 is that while endocytosis is affected, other β-arrestin–mediated functions remain unchanged when examined in β-arrestin KO cells (4, 9). The reason for this remains to be determined. Interestingly, the chemokine receptor CXCR4 also follows a β-arrestin–independent/GRK-dependent mode of endocytosis and recently, sorting nexin 9 was described as an endocytic cargo adaptor for this receptor (5). The sorting nexin 9 family does not appear to be essential for CXCR5 endocytosis (5), suggesting CXCR5 follows another mode of endocytosis. In addition, the protease-activated receptor 1, which also follows a β-arrestin–independent/phosphorylation-dependent mode of endocytosis, may internalize *via* a ubiquitin-dependent process (42). Although we previously provided evidence that receptor ubiquitination may not be required for CXCR5 internalization, we cannot rule out the possibility that CXCR5 follows a ubiquitin-dependent process for endocytosis (4). The precise mode of CXCR5 internalization remains to be elucidated.

Classically, GRKs and β-arrestins act in concert to mediate GPCR desensitization, internalization, and promote signaling for some GPCRs (1). It is emerging that these two protein families can have divergent functions in GPCR regulation and in the context of CXCR5, we show that GRKs, not β-arrestins, are essential for agonist-stimulated internalization. Notably, GRK family members generally show phospho-site selectivity, which can have dramatic implications on GPCR regulation, based on the fact that GRK expression varies from tissue to tissue and in disease states (43). However, our data are consistent with receptor determinants and GRKs and possibly other kinases acting in a redundant fashion to fully regulate CXCR5 signaling. Given that CXCR5 signaling is important in several physiological processes (44, 45), such redundancy would ensure that CXCR5 signaling is appropriately maintained for a proper physiological outcome across various tissues in health and disease.

## Experimental procedures

### Cell culture, reagents, and antibodies

Cell lines were maintained at 37 °C with 5% CO_2_ in Dulbecco’s minimum essential medium (DMEM; Cat# 10-013-CV) from Corning and supplemented with 10% (v/v) fetal bovine serum (Cat# 76419-584) from Avantor on 10-cm tissue culture petri dishes. PEI (Cat# 23966) was from Polysciences. Lipofectamine 2000 (Cat# 11668019) and 0.05% Trypsin-EDTA (Cat# 25-300-062) were from Thermo Fisher Scientific. Poly-L-lysine (PLL, Cat# P1399) was from Sigma Aldrich, and white-walled, clear-bottom, 96-well plates (cat# 655094) were from Greiner Bio-One. All PCR amplifications were performed using Platinum SuperFi DNA polymerase from Thermo Fisher Scientific (Cat# 12351010). HEK293 cells were obtained from ATCC (Cat# CRL-1573) or Microbix Biosystems, Inc (Cat# CVCL-0045). HEK293 cells in which β-arrestins1 and β-arrestin2 have been deleted (DKO) were provided by Dr Asuka Inoue (Tohoku University). HEK293 cells in which GRK2/3/5/6 (ΔQ-GRK) have been deleted were previously described (19).

Antibodies against GRK2 (Cat# 13990-1-AP), GRK5 (Cat# 17032-1-AP), FLAG-tag (DYKDDDDK; Cat# 20543-1-AP), and GAPDH (Cat# 600004-1-Ig) were from Proteintech. Antibody against HA-tag (Cat# 923501) was from BioLegend. The antibody against clathrin heavy chain (CLTC, Cat# sc-12734) was from Santa Cruz Biotechnology. Horse radish peroxidase–tagged secondary anti-rabbit (Cat# PI-1000-1) and anti-mouse (Cat# PI-2000-1) antibodies were from Vector Labs. The FLAG “M2” alkaline phosphatase–conjugated antibody (Cat# A9469) was from Sigma-Aldrich. The phospho-specific CXCR5 polyclonal antibodies against pT367/pS368 (Cat# 7TM0072A) and pT370/p371 (Cat# 7TM0072B) were developed in collaboration with 7TM Antibodies GmbH. Different lots of each antibody were used to generate data for each figure (Figure 1, Figure 2, Figure 3). Compound101 (CMPD101, Cat# 5642) was from Tocris Bio-techne and CCG273441 (Cat# 2750414-35-6) was from MedChemExpress.

### Plasmids

DNA plasmids for FLAG-tagged CXCR5 WT, single and double phosphor-cluster receptor variants [Proximal, Medial, Distal, Prox/Med, Prox/Dis, Med/Dis] (4), and HA-tagged CXCR5-RLuc8 were previously described (32, 46, 47). The plasmids for β-arrestin1-GFP^10^ and β-arrestin2-GFP^10^ were provided by Dr Michel Bouvier (Université de Montréal). The GRK2, GRK5, and GRK5 kinase-dead (K215R) were provided by Dr Jeffrey Benovic (Thomas Jefferson University). The 3×HA-tagged CXCR5 plasmid was a gift from Dr Stefan Schulz (Jena University Hospital). TRUEPATH (Gα_i1_-Rluc8, β3, γ-GFP2) was a gift from Bryan Roth (Addgene kit, #1000000163) (24).

FLAG-tagged CXCR5-CTST [C-terminal region residues S331,S334,T338,T343,S347,S354,S358,S359, S361, S363,T367,S368,T370,T371 were substituted for alanine] was made by PCR amplification of the C-terminal region of the FLAG-Prox/Med variant with overlapping primers to introduce Ala substitutions for Ser/Thr residues in the distal (Dis) region, which was then ligated into a PCR-amplified FLAG-CXCR5 plasmid in which the C-terminal region was excised (G326-F372) using the NEBuilder HiFi DNA Assembly Cloning Kit (NEB, Cat# E5520S) following the manufacturer’s instructions. The GRK2-K220R kinase dead plasmid was made by PCR amplification of GRK2 using nonoverlapping back-to-back primers to introduce an arginine substitution for Lys-220 and then circularized using the Kinase, Ligase, and Dpn1 (KLD) enzyme mix (NEB, Cat# M0554S) according to manufacturer’s instruction. The FLAG-tagged CXCR5-NLuc plasmid was made by PCR amplification of NLuc from the NLuc-EPAC-VV plasmid (48) (kindly provided by Mikel Garcia-Marcos, Boston University, and Kirill Martemyanov, Scripps Florida) with primers that partially overlapped with primers used to amplify the FLAG-CXCR5-pcDNA3 backbone, and PCR products were assembled using the NEBuilder HiFi DNA assembly cloning kit. Plasma membrane–targeted Venus (Venus-CAAX) was made by PCR amplification of Venus with primers that overlapped with primers used to amplify the polybasic membrane targeting sequence of KRas isoform 2B (KKKKKKSKTKCVIM; Uniprot: P01116-2) from previously described Sapphire-PM (5), and PCR products were assembled using the NEBuilder HiFi DNA assembly cloning kit. The early endosome targeted Venus (Venus-2 × FYVE) was made by PCR amplification of Venus with primers that overlapped with primers used to amplify the 2×FYVE domain from a Sapphire-2xFYVE construct, and PCR products were assembled using the NEBuilder HiFi DNA assembly cloning kit. The N-terminally NLuc-tagged β-arrestin1 (NLuc-β-arrestin1) plasmid was made by PCR amplification of the pcDNA3-NLuc backbone with primers that partially overlapped with primers used to amplify a β-arrestin1 from β-arrestin1-GFP^10^ and PCR products were assembled using NEBuilder HiFi DNA assembly cloning kit. The N-terminally NLuc-tagged β-arrestin2 (NLuc-β-arrestin2) plasmid was made by PCR amplification of pcDNA3-NLuc backbone with primers that partially overlapped with primers used to amplify β-arrestin2 from β-arrestin2-GFP^10^ and PCR products were assembled using NEBuilder HiFi DNA assembly cloning kit. All plasmids were sequenced for verification. Clathrin and caveolin1 targeting siRNAs were purchased from Integrated DNA Technologies (https://www.idtdna.com/). A full list of primers and plasmids used in this study is provided in Table S1.

### Detection of receptor phosphorylation

WT HEK293 cells or CRISPR-Cas9 GRK2/3/5/6 quadruple KO (ΔQ-GRK) HEK293 cells grown on 10-cm dishes were transiently transfected at 60 to 70% confluency with 6 μg of either pcDNA (empty vector), 3×HA-CXCR5, FLAG-CXCR5, or FLAG-CXCR5-Distal (Dis) variant using PEI, as previously described (5). For re-expression experiments in the ΔQ-GRK, HEK293 cells were cotransfected with 6 μg of FLAG-CXCR5 and 3 μg of either GRK2, GRK5, or pcDNA. Twenty-four hours later, cells were detached with trypsin and reseeded equally onto two 10-cm dishes and grown overnight. The next day, cells were rinsed with warm DMEM and then serum-starved in DMEM supplemented with 20 mM Hepes (Thermo Fisher Scientific, Cat# 15630080) for 3 h before stimulation with vehicle or 100 nM CXCL13 for 5 to 60 min for time course analysis, or 10 min for other experiments. After stimulation, cells were placed on ice, washed once with ice-cold PBS, and lysed with 500 μl lysis buffer (20 mM Tris–HCl, pH 7.5, 150 mM NaCl, 1% v/v Triton X-100, 1 mM CaCl_2_, protease inhibitor cocktail (Sigma, Cat# P8340) and phosphatase inhibitor cocktails (cocktail 1: Sigma Cat# P2850, cocktail 2: Sigma Cat# P5726; cocktail 3:Sigma Cat# P0044)). Samples were transferred to microcentrifuge tubes, incubated on ice for 10 min, and briefly sonicated on ice followed by centrifugation to pellet the cellular debris at 14,000 rpm using an Eppendorf 5817R tabletop centrifuge for 45 min at 4 °C. Input for each sample was collected and mixed with 2× sample buffer (37.5 mM Tris, pH 6.5, 8% SDS, 10% glycerol, 0.7M BME, and 0.003% (w/v) bromophenol blue). The remaining supernatant was transferred to a fresh microcentrifuge tube and mixed with 20 μl of anti-FLAG M1-agarose affinity beads (Sigma, A4596) (equilibrated 1:1 with lysis buffer) when FLAG-CXCR5 was used or 20 μl of anti-HA agarose affinity beads (Sigma, Cat# E6779) when HA-CXCR5 was used. Tubes were gently rocked for 2 h at 4 °C, followed by rapid centrifugation at 1000 rpm using an Eppendorf 5817R table top centrifuge and three rapid washes with lysis buffer. Samples were eluted in 2× sample buffer and analyzed by 10% SDS-PAGE and immunoblotting onto nitrocellulose membranes. For alkaline phosphatase experiments, immunoprecipitated samples were rapidly washed in calcium-supplemented Tris-buffered saline (TBS, 20 mM Tris–HCl, pH 7.5, 150 mM NaCl, 1 mM CaCl_2_). Then, FLAG peptide (Sigma, Cat# F3290) at 100 μg/ml was added for 15 min to elute FLAG-tagged receptor; samples were divided in half and treated with 5 U of alkaline phosphatase (FastAP, Thermo Fisher Scientific, Cat# EF065) or vehicle for 2 h at 37 °C. Reactions were terminated by adding 2× sample buffer and samples were analyzed by 10% SDS-PAGE and immunoblotting onto nitrocellulose membranes. Membranes were incubated with 5% (v/v) fetal bovine serum in Tris-buffer saline + 0.05% (v/v) Tween-20 (TBST) (RPI, Cat# 9005-64-5) overnight at 4 °C at a 1:500 dilution with anti-pT367/pS368 and anti-pT370/pT371 antibodies. Membranes were then washed 3× with TBST before incubation with anti-rabbit horse-radish peroxidase–conjugated antibody in 5% milk/TBST at room temperature for 1 h. After extensive washing in TBST, membranes were developed with Amersham ECL Prime Western Blotting Detection Reagent (Sigma; Cat# RPM2236) solution for 5 min. Images were acquired on a Chemidoc touch imaging system (Bio-Rad). Membranes were stripped with Restore PLUS Western blot stripping buffer (Thermo Fisher Scientific, Cat# 46430) for 15 min at room temperature and probed for epitope-tagged receptor with anti-FLAG or anti-HA antibodies. Densitometric analysis was performed using ImageJ software.

### BRET-based G protein activation assay

WT HEK293 cells and CRISPR-Cas9 quadruple GRK2/3/5/6 KO HEK293 cells (ΔQ-GRK) cultured in 10-cm dishes were transiently transfected at approximately 60 to 70% confluency with 3 μg of FLAG-tagged CXCR5 and 1 μg each of TRUEPATH constructs (G_αi1_-Rluc8, β3, and γ-GFP_2_) using PEI. After 24 hours, cells were detached with trypsin and reseeded onto PLL-coated 96-well plate at a density of 20,000 cells/well. The next day, cells were washed once with HBSS and incubated with 40 μl of HBSS and 5 μM coelenterazine-400a (Nanolight, Cat# 340-1) and allowed to equilibrate at room temperature for 5 min away from light. Baseline BRET signals were measured for ∼2 min at room temperature with an integration time of 0.1 s using a microplate reader (LUMIstar Omega, BMG Labtech), followed by the manual addition of varying concentrations of CXCL13 (1–100 nM) and the BRET signal was measured every 30 s for a total of 15 min. The BRET ratio was calculated as the emission signal at 515 ± 30 nm divided by the emission signal at 410 ± 80 nm. The average BRET signal of the baseline was subtracted from the BRET of the veh or CXCL13 response and normalized to receptor expression (ΔBRET). The AUC was calculated for each concentration of CXCL13 and then normalized to vehicle condition of the WT HEK293 cells and data fit by a nonlinear regression model [log[agonist] *versus* response (three parameters)] using GraphPad Prism for macOS, GraphPad Software.

### BRET assay for β-arrestin recruitment to CXCR5

WT HEK293 cells and CRISPR-Cas9 quadruple GRK2/3/5/6 KO HEK293 cells (ΔQ-GRK HEK293) cultured in 10-cm dishes to approximately 60 to 70% confluency were transiently transfected with 100 ng of HA-tagged CXCR5-Rluc8 and 300 ng of either β-arrestin1-GFP^10^, β-arrestin2-GFP^10^, or pcDNA with PEI as previously described (4). For experiments where GRKs were re-expressed in ΔQ-GRK HEK293 cells, 50 ng of GRK2, GRK5, or pcDNA were cotransfected with receptor and β-arrestin plasmids. After 24 hrs, cells were detached with trypsin and reseeded into a PLL-coated 96-well plates at around 20,000 cells/well. The next day, cells were washed once with 100 μl of HBSS, then incubated with 80 μl of HBSS and 5 μM coelenterazine-400a (Nanolight, Cat# 340-1) at room temperature for 5 min. After the manual addition of varying concentrations of CXCL13 (1–100 nM), the BRET signal was measured every 30s for 20 min at room temperature with an integration time of 0.1 s in a microplate reader (PHERAstar, BMG Labtech). The BRET ratio was calculated as the emission signal at 515 ± 30 nm divided by the emission signal at 410 ± 80 nm. The net BRET was calculated by subtracting the BRET ratio from cells transfected with only donor plasmid, then the net BRET of the first three reads were averaged together. BRET signals were normalized to the highest CXCL13 dose. All graphs were plotted in GraphPad PRISM using the nonlinear regression model: [log[agonist]vs. response (three parameters)].

For kinetic analysis of β-arrestin recruitment to CXCR5, we used a proximity-based BRET assay that measures β-arrestin recruitment to CXCR5 by detecting BRET with a plasma membrane–targeted bystander protein, similar to what has been used by others (49). These experiments were performed in ΔQ-GRK HEK293 cells grown on 10-cm dishes transiently cotransfected with 50 ng of either GRK2, GRK5, or pcDNA and 150 ng NLuc-β-arrestin1 or NLuc-β-arrestin2 and 2 μg of plasma membrane–targeted Venus (PM-Venus) using PEI. After 24 hours, cells were detached with trypsin and reseeded onto PLL-coated 96-well plates at a density of 20,000 cells/well. The next day, cells were washed once with HBSS and incubated with 40 μl of HBSS and 5 μM furimazine (AOBIOUS, Cat# AOB36539) and after equilibration for 10 min at room temperature, baseline BRET signals were measured for ∼3.5 min at room temperature every 10 s with an integration time of 0.24 s using a microplate reader (PheraStar, BMG Labtech), followed by the manual addition of 100 nM CXCL13 or vehicle and the BRET signal was measured every 10 s for 20 min in total. The BRET ratio was calculated as the emission signal at 515 ± 30 nm divided by the emission signal at 410 ± 80 nm. The average BRET signal of the baseline was subtracted from the BRET values of the vehicle or CXCL13 signal (BRET), and then the vehicle BRET was subtracted from the CXCL13 BRET (ΔBRET). Data were fit against a one-phase association nonlinear regression model, and the AUC was calculated for vehicle and CXCL13 using GraphPad Prism for macOS, GraphPad Software.

### Measurement of cell surface receptor

Receptor internalization was determined as a loss of receptor from the cell surface using whole-cell ELISA as previously described (4, 5). Briefly, WT HEK293 cells or ΔQ-GRK HEK293 cells grown to 60 to 70% confluency on 10-cm dishes were transiently transfected with 6 μg of FLAG-tagged CXCR5 or phospho-site cluster variants with PEI. For experiments where GRKs were re-expressed in ΔQ-GRK HEK293 cells, 3 μg of GRK2, GRK2-K220R, GRK5, GRK5-K215R, or pcDNA were cotransfected with 6 μg of FLAG-tagged CXCR5 WT or variants using PEI. For experiments where siRNA targeting clathrin heavy chain 1 (CLTC) was used, transfections were in β-arrestin1/2 double knockout (DKO) HEK293 cells cultured on 6-cm dishes to 80 to 90% confluency. For these transfections, DNA plasmids (3 μg of FLAG-tagged CXCR5) and siRNA (20 nM final) were cotransfected using Lipofectamine 2000 as we have previously described (50). After 24 hrs cells, cells were detached with trypsin and reseeded into a PLL-coated 24-well dishes at around 200,000 cells/well. Twenty-four hours later, cells were washed once with serum-free DMEM containing 20 mM Hepes and then stimulated with 100 nM CXCL13 or vehicle at 37 °C for 30 min. Dishes were placed on ice and cells washed with ice-cold tris-buffered saline (TBS, 20 mM Tris–HCl, pH = 7.5; 150 mM NaCl) and fixed with 3.7% v/v formaldehyde (Sigma, cat. No. F8775) in TBS for 5 min at room temperature. Cells were washed three times with TBS, followed by a blocking step in which cells were incubated at room temperature for 45 min with TBS containing 1% (w/v) BSA (TBS-BSA). Next, cells were incubated with an anti-FLAG “M2” antibody conjugated to alkaline phosphatase (1:1000 dilution in TBS-BSA) for 60 min at room temperature. After three washes with TBS, cells were incubated with p-nitrophenol phosphate solution (Sigma, cat. No. P7998) until a mild yellow color appeared, and absorbances were measured at 405 nm. Receptor internalization was calculated as a percent decrease in the background-adjusted absorbance from CXCL13-stimulated cells relative to vehicle-treated cells. For experiments with GRK re-expression or siRNA knockdown, whole cell lysates were collected from each transfection in 2 × sample buffer for 10% SDS-PAGE and immunoblotting analysis.

### Bystander BRET to measure receptor internalization

For kinetic analysis of CXCR5 internalization under pharmacological inhibition of GRKs, we used a proximity-based BRET assay that measures internalization as a loss of proximity to a plasma membrane–targeted bystander fluorescent protein and gain of proximity to an early endosome-targeted bystander fluorescent protein, similar to what has been described by others (25). HEK293 cells grown to 60 to 70% confluency in 10-cm dishes were transiently transfected with 100 ng of FLAG-tagged CXCR5 also tagged in-frame with NLuc and either 2 μg of PM-Venus or EE-Venus, and either 2 μg pcDNA or dynamin-K44A. For the GRK rescue, ΔQ-GRK HEK293 cells grown to 60 to 70% confluency in 10-cm dishes were transiently transfected with 100 ng of FLAG-CXCR5-NLuc and either 1 μg of PM-Venus or EE-Venus, and either 1 μg pcDNA, WT GRK2 or GRK5, and kinase dead 1 μg GRK2 or 2 μg GRK5. After 24 hrs, cells were detached with trypsin and reseeded into PLL-coated 96-well plates at approximately 20,000 cells/well. The next day, cells were washed once with HBSS and then incubated with 80 μl of HBSS and 10 μl furimazine (5 μM final). Wells were allowed to equilibrate at 37 °C in a plate reader (Pherastar, BMG Biotech) for 5 to 10 min away from light. After equilibration for 5 min, baseline BRET signals were measured at 37 °C every 30 s with an integration time of 0.1 to 0.2 s for 5 min. After manual addition of 100 nM of CXCL13 or vehicle, the BRET signal was measured every 30 s for a total of 30 min at 37 °C. These BRET measurements were made at 37 °C to facilitate receptor internalization. The BRET ratio was calculated as the emission signal at 515 ± 30 nm divided by the emission signal at 410 ± 80 nm. The average BRET signal of the baseline was subtracted from the BRET of the CXCL13 or vehicle response, and then the vehicle BRET was subtracted from the CXCL13 BRET (ΔBRET). Data were fit by a nonlinear regression model [log[agonist] *versus* response (three parameters)] and the AUC was calculated using GraphPad Prism for macOS, GraphPad Software.

For experiments using kinase inhibitors, HEK293 cells were transiently transfected as described above, except the dynamin-K44A mutant. Cells were detached 24 hrs later and plated in a PLL-coated 96-well plate at ∼20,000 cells/well. Following another 24 hrs, cells were washed once with HBSS. Then cells were incubated with 70 μl HBSS and 10 μl of CMPD101 (10 μM final) or CCG273441 (10 μM final) alone and together, or DMSO for 15 min at 37 °C, followed by the addition of 10 μl of furimazine (5 μM final) to each well. After 5 min of equilibration, baseline BRET values were determined and the addition of vehicle of CXCL13 (100 nM) was carried out as previously described. The average BRET signal of the baseline was subtracted from the BRET of the CXCL13 or vehicle response and then the vehicle BRET was subtracted from the CXCL13 BRET (ΔBRET). Data were fit by a nonlinear regression model [log[agonist] *versus* response (three parameters)] and the AUC was calculated using GraphPad Prism for macOS, GraphPad Software.

### Statistical analysis

All data represents the mean ± SD of at least three or more independent experiments as indicated in the figure legends. Statistical analyses were performed in Prism Software version 10.4.1 (GraphPad). Student’s *t* test was used to compare two groups, and one-way or two-way ANOVA was used to compare multiple conditions, followed by either Dunnett’s or Šídák's multiple comparison test. Specific statistical tests are indicated in the figure legends. *p* values less than or equal to 0.05 were considered significantly different for all comparisons.

## Data availability

All data to support the conclusions of our study are contained within the manuscript and supporting information. All plasmids described in this study are available from the corresponding author upon reasonable request, subject to a completed Materials Transfer Agreement.

## Supporting information

This article contains supporting information.

## Conflict of interest

The authors declare that they have no conflicts of interest with the contents of this article.

## Supporting information

Table S1

Plasmid Table (Table S1)
