# Supplementary material for: GRK phosphorylation drives β-arrestin–independent internalization of chemokine receptor CXCR5
Source: J Biol Chem. 2025 Dec 29;302(2):111114. doi: 10.1016/j.jbc.2025.111114 (PMC12859505; doi:10.1016/j.jbc.2025.111114)
Supplement: Supporting Information [file mmc1.docx]

**GRK phosphorylation drives β-arrestin-independent internalization of chemokine receptor CXCR5**

by

Joseph M. Crecelius*, Ya Zhuo*, Aaren R. Manz*, Julia Drube^***^, Stefan Schultz^**^, Carsten Hoffmann^***^, and Adriano Marchese^*^

*Department of Biochemistry, Medical College of Wisconsin, Milwaukee WI 53226

**Department of Pharmacology and Toxicology, Jena University Hospital, Germany and 7TM Antibodies, Jena, Germany

*** Institut für Molekulare Zellbiologie, CMB – Center for Molecular Biomedicine; Universitätsklinikum Jena; Friedrich-Schiller-Universität Jena; Hans-Knöll Straße 2, D-07745 Jena; Germany

Supporting Information Included:

Figure S1

Figure S2


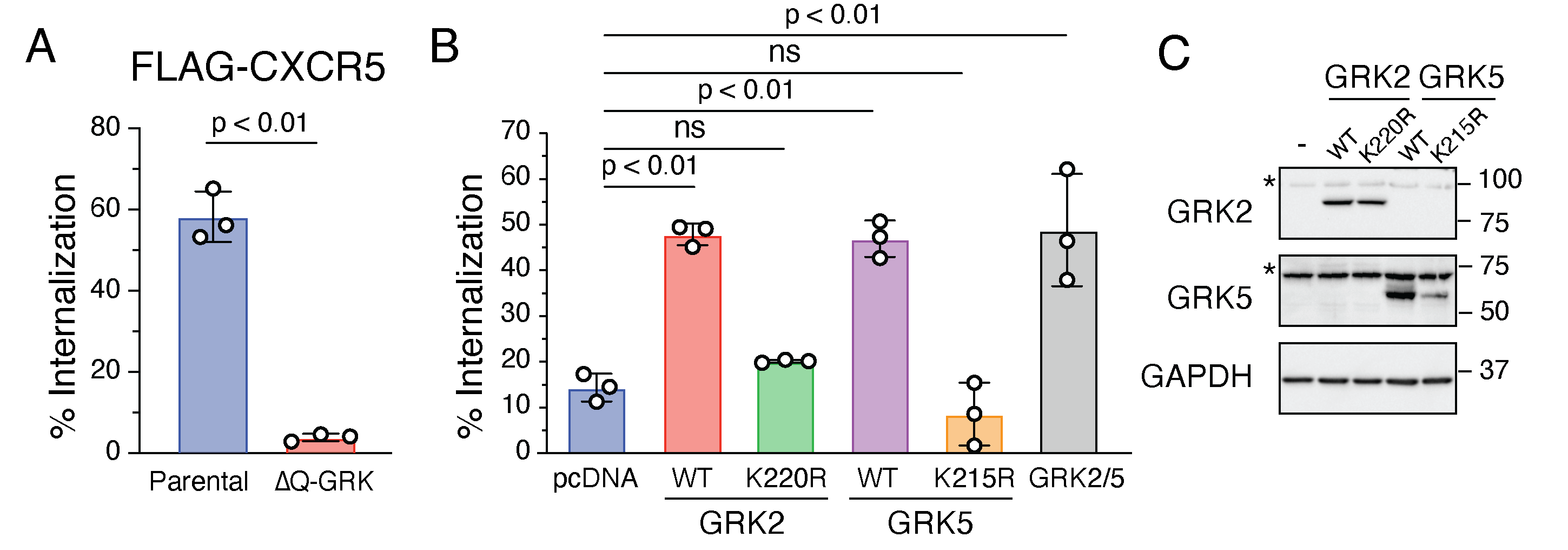


**Figure S1. GRKs are essential for agonist-stimulated internalization of CXCR5 as assessed by ELISA.** **A-B**. Parental or ∆Q-GRK HEK293 cells transiently transfected with FLAG-CXCR5 (A) and ∆Q-GRK HEK293 cells transiently expressing FLAG-CXCR5 or either empty vector (pcDNA), wild-type (WT) GRK2 and GRK5 or together (GRK2/5) or kinase-dead mutants of GRK2 (K220R) and GRK5 (K215R) (B). Cells were stimulated without (vehicle) or with 100 nM CXCL13 for 30 min at 37°C. Cell surface receptor was measured by whole-cell ELISA with an anti-FLAG antibody conjugated to alkaline phosphatase (M2-AP), as described in *Experimental procedures*. Internalization was calculated as a percent decrease in the background-adjusted absorbance values from CXCL13-stimulated cells relative to vehicle-treated cells. Data represent the mean ± S.D. from 3 independent experiments. Data were compared by unpaired Student’s T-test (A) or one-way ANOVA with Dunnett’s multiple comparison test (B). P values are indicated; ns = not significant. **C.** Representative immunoblots with indicated antibodies are shown. The asterisk (*) represents a non-specific band.

**Figure S2. Surface expression of CXCR5 as assessed by ELISA.** **A-B**. ∆Q-GRK HEK293 cells were transiently transfected with FLAG-tagged wild-type (WT) and single phospho-site cluster variants (A) or double phospho-site variants plus empty vector (pcDNA), GRK2 and GRK5 (B). Cell surface receptor was measured by whole-cell ELISA with an anti-FLAG antibody conjugated to alkaline phosphatase (M2-AP), as described in *Experimental procedures*. Surface expression was calculated relative to the WT receptor and pcDNA-transfected cells. These data are from the vehicle condition of the data reported in Figure 9, panels B and C. Data represent the mean ± S.D. from 3 independent experiments. Data were compared by one-way ANOVA with Dunnett’s multiple comparison test. The asterisk (*) represents adjusted P value < 0.05 compared to WT receptor and pcDNA, while all other comparisons were not significant. Cell surface levels of each receptor are similar across all respective transfections.
