# Supplementary material for: GRK phosphorylation drives β-arrestin–independent internalization of chemokine receptor CXCR5
Source: J Biol Chem. 2025 Dec 29;302(2):111114. doi: 10.1016/j.jbc.2025.111114 (PMC12859505; doi:10.1016/j.jbc.2025.111114)
Supplement: Table S1 [file mmc2.docx]

**Supplemental Table S1. Plasmid Table.**

List of new plasmids described in this study. The backbone and fragment used for Gibson assembly were generated by PCR using overlapping primers (overlapping nucleotides indicated in blue font) and assembled with HiFi DNA Assembly kit (NEB, E5520S). All other constructs were generated by PCR using back-to-back primers and circularized with kinase, ligase, and Dpn1 (KLD) enzyme kit (NEB, M0554S). For KLD reaction of GRK-K220R, since there was no second template, it is listed as non-applicable (N/A). Amino acid residues that were mutated are indicated. The sequence of primers is indicated with the forward primer (For) above and the reverse primer (Rev) below.
